# ‘Building the atomic clinic’: nuclear research in medicine after the Second World War – an introduction

**DOI:** 10.1017/mdh.2026.10086

**Published:** 2026-10

**Authors:** Johannes Mattes, Cécile Philippe, Maria Rentetzi

**Affiliations:** 1 Division of Nuclear Medicine, Department of Biomedical Imaging and Image-guided Therapy, Medical University of Vienna, Vienna, Austria; 2 Department of Research Library and Reflection on Science, National Academy of Sciences Leopoldina, Halle (Saale), Germany; 3 Chair of Science, Technology, and Gender Studies, Friedrich-Alexander-Universitat Erlangen-Nurnberg, Erlangen, Germany

## Abstract

This introduction to the special issue re-examines the emergence of nuclear medicine within a global and transdisciplinary framework. Moving beyond canonical narratives centred on technical breakthroughs and early U.S. developments, it highlights the field’s development through three analytical lenses: science diplomacy, transdisciplinarity, and socio-political impact. We argue that nuclear medicine took shape not only through innovations in physics and clinical practice, but also through international cooperation, geopolitical agendas, and the negotiation of public expectations. By tracing the circulation of knowledge, radioisotopes, and infrastructure across diverse regional contexts – from Sweden and Taiwan to India and Belgium – it sets the stage for a rethinking of nuclear medicine as a product of intersecting scientific, political, and societal forces during the Cold War and beyond.

‘Could iodine be made radioactive artificially?’ wondered Saul Hertz (1905–1950), chief of the Thyroid Unit at Massachusetts General Hospital (MGH), during Karl Compton’s guest lecture on ‘What Physics Can Do for Biology and Medicine’ at Harvard Medical School. It was 12 November 1936. A month later, Compton (1887–1954) – then professor of nuclear physics and president of the Massachusetts Institute of Technology (MIT) – wrote back to Hertz, confirming that indeed ‘iodine can be made artificially radioactive’.^1^ At the time, Hertz was investigating the role of iodine in thyroid metabolism and its potential for therapeutic use. Compton’s affirmative response led to a collaborative project between MGH and MIT, bringing together Hertz, Compton, and several early‐career physicists then investigating the widely publicised case of radium-dial painter poisoning. In this U.S. industrial scandal, young women had ingested dangerous amounts of radium by shaping paintbrushes with their lips, resulting in grave health consequences and increased public and regulatory concern over radiation exposure.^2^ Drawing on lessons from this case, Robley Evans (1907–1995) and Arthur Roberts (1912–2004) teamed up with Hertz to develop safe and reliable methods for producing and applying radioiodine in clinical contexts.^3^

Meanwhile, on the West Coast, another hub for exploring the medical potential of radioisotopes had emerged at the University of California, Berkeley. There, physician John Lawrence (1904–1994) joined forces with his brother, physicist Ernest Lawrence (1901–1958), later a Nobel laureate.^4^ Together, they investigated the biochemical and physiological effects of radioactive substances generated by Ernest’s newly patented cyclotron, a novel type of particle accelerator that enabled the systematic production of radioisotopes for experimental and therapeutic use. In 1936, John was appointed head of the university’s medical research laboratory, later known as the Donner Laboratory.^5^ In this role, he conducted radiation studies on humans, including the injection of radioactive phosphorus-32 into leukaemia patients – experimental procedures now considered ethically controversial. Shortly thereafter, the Lawrence brothers expanded their work to cancer treatment using neutron beams.^6^

A decade later, in 1946, the headquarters of the U.S. Manhattan Project, operating the world’s first experimental nuclear reactors, issued a public announcement on the availability of radioactive isotopes, published in the prestigious journal *Science*: ‘Production of tracer and therapeutic radioisotopes has been heralded as one of the great peacetime contributions of the uranium chain-reacting pile. This use of the pile will unquestionably be rich in scientific, medical, and technological applications’.^7^ In retrospect, this statement not only signalled the dawn of the modern radioisotope era but also marked the beginning of the wider integration of nuclear technologies into healthcare following the Second World War. Just a year later, the reactor at Oak Ridge National Laboratory in Tennessee – the nation’s primary isotope production site – had already shipped 407 orders of radioactive materials for therapeutic use; by 1952, that number had increased tenfold.^8^

Reflecting on this formative phase, Baird G. Whaley (b. 1929), long-time administrator at the Donner Laboratory, recalled that the emerging field at the crossroads of physics and medicine was initially referred to as ‘medical physics’.^9^ Another, though inconsistently used, label was ‘atomic medicine’, which also encompassed the treatment of radiation-induced harm.^10^ According to Whaley, by the mid-1950s, as experimental techniques entered hospital practice and clinicians became more actively involved,

… they [the Berkeley group] were beginning to call it ‘nuclear medicine’… I think a lot of it had to do with politics and power. Medical physics was coming to be seen as a very narrow part of the field. They preferred it to be called ‘biophysics,’ because that was a more encompassing field. For the strictly medical end, they liked ‘nuclear medicine’ better, as terminology. And that was the name of their professional society. It was the ‘Society of Nuclear Medicine’ [established in 1956 as a national organisation], not the ‘Society of Medical Physics’.^11^

A further step in consolidating the new term came in 1952, when it was incorporated into the title of the *American Journal of Roentgenology, Radium Therapy and Nuclear Medicine*, followed by similar adjustments in European periodicals.^12^ At the time, nuclear medicine referred to a still largely experimental set of therapies that used artificially produced radioisotopes from reactors or cyclotrons, administered into the body, to treat oncological, orthopaedic, and related conditions. This distinguished it from earlier clinical uses of X-rays and naturally occurring radium, typically applied externally.^13^ Given overlaps in practitioners, physical principles, and areas of application, the emerging field was framed as an extension of radiology and internal medicine.^14^

Between 1955 and 1965, the scope of nuclear medicine began to shift as a series of innovations expanded its diagnostic potential. Central to this transformation was the use of radioactive tracers, allowing in vivo tracking of physiological processes and visualisation of organ function.^15^ At the same time, the introduction of technetium-99m as a versatile radioisotope, along with portable technetium generators, facilitated clinical use – even in regional settings.^16^ Advances in imaging technology, from Benedict Cassen’s rectilinear scanner (1950) to Hal Anger’s scintillation (gamma) camera (1957), enhanced the localisation of pathological changes.^17^ In parallel, mathematical models like compartmental analysis improved understanding of circulatory and metabolic functions, while Rosalyn Yalow’s invention of the radioimmunoassay (1959/60) expanded in-vitro diagnostic capabilities.^18^

Yet this initial boom proved short-lived. With the rise of external beam radiotherapy and chemotherapy, many treatments involving ingested or injected radioisotopes were gradually abandoned, with only a few, notably for thyroid and blood cancers, remaining in routine use.^19^ Likewise, morphological visualisation techniques such as sonography and computed tomography (CT) displaced nuclear medicine from its central role in medical imaging by the 1970s. Subsequently, the field shifted its focus to in vivo diagnostics and the functional analysis of metabolic processes, with thyroid disorders – especially those assessed through early standardised radioiodine uptake tests – remaining a central area of application.^20^ By the 2000s, nuclear medicine had transitioned to molecular imaging. Theranostics, linking diagnostic and therapeutic approaches, have emerged as significant clinical innovations, particularly in oncology.^21^ Meanwhile, the pharmaceutical industry’s growing role in developing radiopharmaceuticals has shaped research priorities and integrated the field into commercial frameworks. Most recently, hybrid imaging devices such as positron emission tomography (PET) combined with CT have blurred the lines between nuclear medicine and radiology, prompting new institutional collaborations and disciplinary redefinitions.^22^

## Origins and identity: narratives defining nuclear medicine

As nuclear medicine expanded in scope and application, so too did the narratives that sought to articulate its origins and identity. A widely accepted account traces the field’s beginnings to the 1920s, crediting Hungarian chemist George de Hevesy (1885–1966) – renowned for his Nobel Prize-winning work on the use of radioactive isotopes as biological tracers – as the ‘founding father’ of nuclear medicine.^23^ Other accounts highlight figures such as the aforementioned Saul Hertz and John Lawrence, who pioneered therapeutic applications of radioisotopes in the 1930s.^24^ Much of the early historiography emerged from within the field itself, authored by eminent practitioners drawing on personal experience and institutional memory, often in the context of commemorative publications.^25^ These narratives tend to focus on developments in the Anglo-American world, especially early U.S. laboratory and hospital settings, and concentrate on the formative period up to 1950. Within this framework, the Second World War appears as a pivotal moment: it accelerated the circulation and commodification of radioisotopes and provided the infrastructure and momentum for later advances in radiopharmaceuticals and diagnostic imaging.

While this early historiography laid essential groundwork, we collectively argue that a more comprehensive understanding of nuclear medicine must also account for its development and global spread during the Cold War. To this end, we adopt a broad conception of the field – one that considers not only its clinical scope in the 1940s and 1950s or its present-day applications, but also the wider political and social contexts of its emergence. It was only in the early 1960s that practitioners began to assert nuclear medicine as ‘a discipline in its own right’, marked by the creation of dedicated university chairs and hospital departments, the appearance of specialised textbooks, and the worldwide adoption and commercialisation of nuclear imaging technologies.^26^ Early centres emerged not only at Massachusetts General Hospital (Boston) and Hammersmith Hospital (London), but also the Service Hospitalier Frédéric-Joliot (Orsay), Bonn University Hospital, and Hadassah Hospital (Jerusalem).^27^ This institutional growth was soon accompanied by technical assistance missions launched by the International Atomic Energy Agency (IAEA). This UN-related organisation, founded in 1957 and headquartered in Vienna, aimed to establish clinical isotope laboratories in East Asia, Latin America, and the Middle East.^28^ During this formative period, medical radioisotope applications – situated at the nexus of radiology and physiology, laboratory research and hospital care – were profoundly shaped by power-political tensions, public hopes and fears surrounding nuclear technologies, and stark inequalities in access to resources and expertise, particularly in low- and middle-income countries. Therapeutic efforts in oncology, such as whole-body irradiation and isotope-based cancer treatments, were often linked to military research agendas, especially in the early Cold War, when they were embedded in broader visions of nuclear society and civil defence.^29^

Our argument draws on a growing body of scholarship that examines the role of researchers in international affairs and the impact of science and technology on global governance.^30^ This literature has analysed large-scale scientific initiatives, multilateral organisations, and transatlantic exchanges during the Cold War, highlighting the complexities of scientific diplomacy, the standard-setting functions of international agencies, and the material culture of nuclear negotiations.^31^ In parallel, local and national case studies, ranging from radioiodine therapy to the interweaving of governmental, industrial, and clinical agendas, demonstrate how global technological developments and regionally specific healthcare practices were mutually shaped and co-produced.^32^

## From origins to global entanglements: diplomacy, disciplines, and power

In this special issue, the authors delve into the historical use of radioisotopes in medicine, tracing their social and political reverberations to illuminate how knowledge, technologies, and practices evolved, circulated, and resonated across diverse healthcare systems and governance regimes. Moving beyond canonical accounts focused on technical breakthroughs and institutional milestones, we reframe the history of nuclear medicine by highlighting broader dynamics such as scientific internationalism, hospital-industry collaborations, and global health initiatives – while also scrutinising the tensions, power struggles, and inequalities embedded within these processes. This perspective shows how shifts in therapeutic and diagnostic applications were entangled with standardisation efforts that often reflected and reinforced wider political and social hierarchies. It also uncovers the roles of key actors across disciplinary and institutional boundaries, the rise of multilateral organisations and exchange mechanisms, and the far-reaching effects of technological and clinical innovation. Over the course of assembling this volume, three critical dimensions emerged as central analytical themes: (a) science diplomacy, (b) transdisciplinarity, and (c) socio-political implications – each addressed with varying emphasis in the individual contributions. Through this lens, we seek to deepen understanding of the complex interplay between nuclear research in medicine, geopolitics, and societal change.

First, adopting a science diplomacy perspective highlights the international scope of nuclear medicine and its uneven development after 1945. This global trajectory was shaped by U.S. and U.K. leadership in radioisotope production and application, alongside the creation of multilateral institutions, most notably the IAEA and the World Health Organization (WHO). Cross-border cooperation – including training programmes for medical professionals and efforts to standardise hospital practice and instrumentation – proved central to the worldwide dissemination of radioisotope technologies. The targeted provision of nuclear technologies to low- and middle-income countries, particularly across the Global South, further facilitated their incorporation into local medical infrastructures.

Second, approaching nuclear medicine as a transdisciplinary enterprise – merging basic research, clinical practice, and engineering – brings into focus the complex dynamics of collaboration and the roles of diverse experts in shaping this medical speciality.^33^ The development of new radiopharmaceuticals and therapeutic applications, for instance, depended on the combined expertise of physicians, physicists, chemists, and other specialists. At a stage when clinical radioisotope applications were still relatively new and not yet fully professionalised, this cross-disciplinary framework could open new career pathways for women scientists and help to expand their presence in the traditionally male-dominated sphere of clinical medicine.^34^ At the same time, it generated professional tensions, especially among overlapping specialities such as radiology and radiotherapy.

Third, examining the socio-political implications of nuclear medicine reveals the persistent tensions between high infrastructure costs, public expectations, and the priorities of medical professionals. These dynamics have profoundly shaped the clinical use of radioisotopes – at once celebrated as symbols of technological progress and scrutinised as subjects of public debate.^35^ Ongoing concerns over their actual benefits, issues of equitable access, and the ethical responsibilities tied to research and application continue to shape both the practice and perception of nuclear technologies in medicine. Such challenges underscore the necessity of situating the field and its practitioners within broader political economies and global power structures, revealing how technological advancements are inseparable from questions of social justice and international relations.

The articles in this special issue do not seek to provide a comprehensive history of nuclear research in medicine. Rather, they examine selected episodes and trajectories that illuminate the key dimensions outlined above. Central questions include: How have nuclear medicine practices evolved across different geopolitical settings? What roles have multilateral organisations, national policies, and local public health priorities played in shaping their trajectories? And how have technological infrastructures and social dynamics intersected to redefine the applications and expectations of nuclear medicine? The articles embed these questions within broader historical transformations – marked by war, the disintegration and reconfiguration of empires, and the rise of U.S. scientific hegemony. Instead of focusing solely on prominent physicians and institutions, the volume highlights intermediary and local actors operating across clinical, technical, and diplomatic domains. Political change, in some cases, served as a catalyst for scientific reorganisation and institutional innovation. The uneven global uptake of radioisotope applications reflects not only colonial legacies and Cold War agendas but also their selective integration into locally embedded clinical expertise. As nuclear medicine technologies became entwined with postcolonial modernisation projects and, later, market-oriented industrial strategies, they were reshaped by tensions between public health needs, global inequalities, and the commercialisation of science. Taken together, the contributions to this special issue move beyond conventional narratives to reframe nuclear medicine as a field forged at the intersection of shifting political orders, technological regimes, and social imaginaries – offering critical insight into both its historical constitution and its ongoing transformation.

## From Sweden to Taiwan and from India to Belgium: nuclear medicine in the making

In the first paper, Aske Hennelund Nielsen examines the contributions of the Swedish medical physicist Rolf Sievert (1896–1966) to the development of international radiation protection, with particular emphasis on radiological medicine, standardisation, and safety. Best known today for the radiation dose unit named in his honour, Sievert is portrayed as a ‘diplo-scientific actor’ – a figure whose work combined medical physics, radiological practice, and international scientific diplomacy. The paper traces Sievert’s influence on the International Commission on Radiation Protection (ICRP), from its foundation in 1928 to his critical post-war efforts to reorient its mission in response to the realities of the nuclear age.

Nielsen further highlights Sievert’s enduring commitment to radiological medicine, beginning in the 1920s with his work on standardising the clinical use of X-rays and radium. Throughout his career, Sievert sought to balance the therapeutic and diagnostic benefits of radiation with the imperative of limiting risks to patients and healthcare workers. After the Second World War, his focus broadened to questions of public exposure, as nuclear technologies proliferated and concerns over radioactive fallout gained prominence. His advocacy proved instrumental in expanding the ICRP’s remit from medical and radiological settings to wider populations and broader questions of public health. By centring Sievert’s medical-radiological background and sustained engagement with radiation protection, Nielsen shows how his efforts helped transform radiation safety from a specialised professional concern into a matter of global health and risk governance.

Hsiu-Yun Wang takes us to Cold War Taiwan and examines the introduction and limited adoption of iodine-131 (radioactive iodine) as a treatment for hyperthyroidism, situating this medical development within its broader geopolitical, colonial, and cultural contexts. While iodine-131 was hailed as a revolutionary therapy for thyroid disease in the United States after the Second World War, its integration into Taiwan’s medical system faced considerable obstacles. Wang argues that the promotion of radioisotopes in Taiwan formed part of U.S. efforts to extend its influence in Asia through the Atoms for Peace programme, positioning nuclear technologies as instruments of Cold War diplomacy. This initiative aligned with the Republic of China’s nation-building efforts under U.S. patronage, framing nuclear medicine as both a symbol of modernity and an anti-Communist counterpoint to the People’s Republic of China.

However, Taiwan’s response was shaped by enduring medical traditions rooted in its Japanese colonial past. Thyroidectomy – a surgical treatment for hyperthyroidism – had been widely practised during colonial rule and remained dominant after 1945, supported by a well-established surgical community and institutional infrastructure. In contrast, the use of radioisotopes required costly equipment, imported materials, specialised expertise, and reliable access to iodine-131, limiting its availability to elite institutions such as National Taiwan University Hospital. Furthermore, the high cost of treatment rendered it inaccessible to much of the population. Wang’s analysis underscores that the global diffusion of medical innovations depends not only on technological advancement or diplomatic promotion, but also on local practices, material conditions, regulatory frameworks, and cultural perceptions. The introduction of clinical radioisotope applications in Taiwan, Wang shows, was shaped by a complex interplay of U.S. Cold War diplomacy, domestic healthcare structures, colonial medical legacies, and competing visions of ‘modernity’.

Kapil Patil’s contribution examines how nuclear medicine emerged in postcolonial India in close connection with the country’s atomic energy programme. Focusing on the decades after independence, the article argues that nuclear medicine was neither a straightforward transfer of ‘Western’ medical technology nor a misplaced project of modernisation. Rather, its development was fundamentally technopolitical, shaped by the interplay of India’s nuclear establishment, the professional ambitions of its radiological community, state-mediated healthcare modernisation, and Cold War networks of scientific aid and assistance. Instead of spreading broadly across the healthcare system, nuclear medicine became concentrated in a small number of metropolitan centres, reflecting both international norms of advanced medical specialisation and the structural limits of India’s medical-industrial capacity.

At the centre of Patil’s analysis is the Radiation Medicine Centre (RMC) at Bombay’s Tata Memorial Hospital, established in 1963 and envisaged by the influential physician and nuclear scientist Homi Bhabha (1909–1966) as part of a broader effort to create a ‘mini-Mayo Clinic’ in India. Supported by international expertise, training, and IAEA and WHO capacity-building initiatives, the RMC became both a clinical site and a laboratory for adapting radioisotope techniques to India’s regional disease patterns. Its work covered a broad spectrum of diagnostic and therapeutic applications, from thyroid disorders and nutritional deficiencies to liver diseases and anaemia, alongside related clinical work in northern India’s ‘goitrous belt’. Patil shows that the consolidation of nuclear medicine in India marked a convergence of professional ambitions, technocratic state-building, and Cold War internationalism, but also remained constrained by outdated instrumentation, limited industrial capacity, and unresolved questions of cost and equitable access.

Who drives radioisotope production in the post-Cold War period – nuclear research centres, processing institutes, clinicians, or the pharmaceutical industry – and how do these stakeholders define the role of radioisotopes in present-day medicine? Hein Brookhuis explores these interrelated questions in his study of research reactors and radiopharmaceutical production in Belgium between 1990 and 2020. He shows how the Belgian Nuclear Research Centre, originally built around nuclear science and energy-related programmes, gradually realigned its mission in response to the growing demands of nuclear medicine and the pharmaceutical industry. Brookhuis argues that this shift was shaped by market forces, public expectations, and political debates over the future of nuclear research.

From the 1990s onwards, molybdenum-99, which decays to produce technetium-99m for diagnostic imaging, remained essential to routine clinical practice, while therapeutic radioisotopes such as lutetium-177 became increasingly important in oncology. Their continued availability not only sustained vital areas of healthcare but also reinforced political and public arguments for preserving and modernising ageing nuclear infrastructure, including Belgium’s BR-2 reactor in Mol. Brookhuis highlights the tensions and opportunities that arose when public research centres became more directly involved in radiopharmaceutical production. Partnerships with pharmaceutical firms – exemplified by the actinium-227/radium-223 supply chain for cancer therapy – opened up potentially lucrative avenues while exposing frictions between state-run institutions and private industry. These developments, he argues, mirror wider transformations in the societal role of ‘big science’. Placing the Belgian experience in a global perspective, Brookhuis ultimately presents radioisotopes as ‘tracers’ not only of biomedical innovation, but also of the broader historical and political forces that shaped nuclear research in medicine.

Together, these contributions reveal that nuclear medicine arose not merely from technical and clinical breakthroughs, but through the interplay of governmental and military agendas, international collaborations, and public discourse. Rather than attributing its origins to a singular ‘founding father’, this special issue gathers diverse global perspectives and narratives that illuminate the field’s complex foundations, while also highlighting persistent gaps and contested interpretations that invite further scholarly attention. These include situated and comparative viewpoints beyond the United States, Western Europe, and major multilateral organisations; social and cultural perceptions of nuclear technologies; ethical questions and patient-centred approaches; as well as economic factors – particularly the shift from publicly to privately funded healthcare and industrial research. Collectively, the authors of this volume argue that nuclear medicine developed at the intersection of established disciplines such as physics and medicine, where engineering meets hospital practice, and within broader arenas of diplomatic negotiation and societal debate. To fully grasp its trajectory and ongoing transformations, it is essential to consider how nuclear medicine has been shaped by political economies and asymmetries of power that continue to define the global landscape of science and health.

## Footnotes

1

Bennett S. Greenspan , Michael S. Hofman , and John Buscombe , ‘The Accomplishments and Legacy of Saul Hertz’, Journal of Nuclear Medicine, 65, 4 (2024), 659–6310.2967/jnumed.124.26752438453358.

2 This scandal unfolded primarily in the late 1910s and early 1920s, when young women employed to paint watch and military instrument dials with radium-based paint commonly shaped their brushes with their lips to achieve precision. The resulting illnesses and deaths sparked widespread media attention and became a landmark case in occupational health and radiation safety. See, for example, Claudia Clark , Radium Girls: Women and Industrial Health Reform, 1910–1935 (Chicago: University of Chicago Press, 1997)11623540; Maria Rentetzi , ‘The Women Radium Dial Painters as Experimental Subjects (1920–1990) or What Counts as Human Experimentation’, NTM Zeitschrift für Geschichte der Wissenschaften, Technik und Medizin, 12, 3 (2004), 233–4810.1007/s00048-004-0201-315584171; and Nicole E. Martinez , Derek W. Jokisch , Lawrence T. Dauer , Keith F. Eckerman , Ronald E. Goans , John D. Brockman , Sergey Y. Tolmachev , Maia Avtandilashvili , Michael T. Mumma , John D. Boice Jr. , and Richard W. Leggett , ‘Radium Dial Workers: Back to the Future’, International Journal of Radiation Biology, 98, 4 (2022), 750–6810.1080/09553002.2021.1917785PMC1056380933900890.

3

Robley Evans , ‘Early History of Nuclear Medicine in Thyroid Studies at Massachusetts General Hospital’, Medical Physics, 2, 3 (1975), 105–910.1118/1.5941761102906; and Frederic Fahey , Frederick Grant , and James Thrall , ‘Saul Hertz, MD, and the Birth of Radionuclide Therapy’, EJNMMI Physics, 4 (2017), 15 10.1186/s40658-017-0182-7PMC540739328451906.

4

Peter Galison and Bruce Hevly (eds), Big Science: The Growth of Large-Scale Research (Stanford: Stanford University Press, 1992); and John Heilbron and Robert Seidel , Lawrence and His Laboratory: A History of the Lawrence Berkeley Laboratory (Berkeley: University of California Press, 1989).

5

Jeffrey E. Williams , ‘Donner Laboratory: The Birthplace of Nuclear Medicine’, Journal of Nuclear Medicine, 40, 1 (1999), 16N–20N 9935047; and Thomas Budinger , Fifty Years of Progress 1937–1987. Donner Laboratory (Berkeley: U.S. Department of Energy, 1987).

6

Hans Svensson and Torsten Landberg , ‘Neutron therapy – The Historical Background’, Acta Oncologica, 33, 3 (1994), 227–3110.3109/028418694090984128018350.

7

Headquarters, Manhattan Project , ‘Availability of Radioactive Isotopes’, Science, 103, 2685 (1946), 697–70520985147, on 697.

8

Becker David and Clark Sawin , ‘Radioactive and Thyroid Disease: The Beginning’, Seminars in Nuclear Medicine, 26, 3 (1996), 155–6410.1016/s0001-2998(96)80020-18829276. On isotope usage and the rise of a radiopharmaceutical market, see Angela N. Creager , Life Atomic: A History of Radioisotopes in Science and Medicine (Chicago: University of Chicago Press, 2015); Alison Kraft , ‘Between Medicine and Industry: Medical Physics and the Rise of the Radioisotope 1945–65’, Contemporary British History, 20, 1 (2006), 1–35 ; and Néstor Herran , ‘Isotope Networks: Training, Sales and Publications’, Dynamis, 29 (2009), 285–306 .

9 United States Department of Energy (ed.), *Human Radiation Studies: Remembering the Early Years. Oral History of Donner Lab Administrator Baird G. Whaley, August 15, 1994*, 5 (Report number: DOE-EH 0479). https://inis.iaea.org/records/pfkgp-e9228 (accessed on 1 May 2025).

10 See, for example, the textbook *Atomic Medicine*, edited by Charles Behrens, E. Richard King, and James Carpender (Baltimore: Williams & Wilkins, 1949), which went through five editions by 1969.

11 United States Department of Energy, *op. cit.* (note 9), 5. On the Society’s history, see C. Craig Harris, ‘The Formation and Evolution of the Society of Nuclear Medicine’, *Seminars in Nuclear Medicine* 26, 3 (1996), 180–90.

12 See the editorial ‘Addition to the Title of the Journal’, *American Journal of Roentgenology, Radium Therapy and Nuclear Medicine* 67, 1 (1952), 114. In today’s terms, radiology (then termed roentgenology) relies on artificially produced X-rays, a type of radiation discovered by Wilhelm Röntgen in 1895, while nuclear medicine uses nuclear radiation, occurring both naturally and artificially, first observed by Henri Becquerel in 1896.

13 On the clinical application of X-rays and radium, see Bettyann Kevles , Naked to the Bone: Medical Imaging (New Brunswick, NJ: Rutgers University Press, 1997); Richard Gunderman , X-Ray Vision: The Evolution of Medical Imaging (Oxford: Oxford University Press, 2013); Charles Hayter , ‘The Clinic as Laboratory: The Case of Radiation Therapy’, Bulletin of the History of Medicine, 72 (1998), 663–8810.1353/bhm.1998.02139879123; and Aimee Slaughter, ‘Harnessing the Modern Miracle: Physicists, Physicians, and the Making of American Radium Therapy’ (unpublished PhD thesis: University of Minnesota, 2013).

14 See Johannes Mattes and Cécile Philippe , ‘Crossing Boundaries, Forging Unity: Nuclear Medicine and Science Diplomacy in Cold War Europe’, The British Journal for the History of Science, 58, 3 (2025), 503–2710.1017/S000708742500031740340850.

15 Radioactive tracers are radioisotopes or radiolabelled compounds that, once introduced into the body, take part in metabolic processes and thereby enable a broad range of diagnostic investigations. For the development of key technologies, see Ralph McCready , ‘Milestones in Nuclear Medicine’, European Journal of Nuclear Medicine, 27, suppl. 1 (2000), S049–79 10.1007/s00259005051810654157.

16 See H. Saul Winchell , ‘Radiopharmaceuticals’, in Henry Wagner (ed.), Nuclear Medicine (New York: HP Publishing, 1975), 61–72 ; and Powell Richards , Walter Tucker , and Suresh Srivastava , ‘Technetium-99m’, International Journal of Applied Radiation and Isotopes, 33 (1982): 793–910.1016/0020-708x(82)90120-x6759417.

17 See Benedict Cassen , Lawrence Curtis , Clifton Reed , and Raymond Libby , ‘Instrumentation for ^131^I Use’, Nucleonics, 9 (1951), 46–50 ; and Hal Anger , ‘Scintillation Camera’, Review of Scientific Instruments, 29 (1958), 27–33 .

18 See Rosalyn Yalow , ‘Development and Proliferation of Radioimmunoassay Technology’, Journal of Chemical Education, 76 (1999), 767–8.

19 See, among others, Jacob van Dyk , Jerry Battista , and Peter Almond , ‘A Retrospective of Cobalt-60 Therapy’, Medical Physics International, 4 (2020), 327–50; and Ellen Leopold , Under the Radar: Cancer and the Cold War (New Brunswick, NJ: Rutgers University Press, 2009).

20 See, for example, Fred Mettler , Arvis G. Williams , James H. Christie , Robert D. Moseley , and Charles A. Kelsey , ‘Trends and Utilization of Nuclear Medicine in the United States: 1972–1982’, Journal of Nuclear Medicine, 26 (1985), 201–53881568.

21 For recent developments, see Wolfgang A. Weber , Johannes Czernin , Carolyn J. Anderson , Ramsey D. Badawi , Henryk Barthel , Frank Bengel , Lisa Bodei , Irène Buvat , Marcelo DiCarli , Michael M. Graham , Jan Grimm , Ken Herrmann , Lale Kostakoglu , Jason S. Lewis , David A. Mankoff , Todd E. Peterson , Heinrich Schelbert , Heiko Schöder , Barry A. Siegel , and H. William Strauss , ‘The Future of Nuclear Medicine, Molecular Imaging, and Theranostics’, Journal of Nuclear Medicine, 61, suppl. 2 (2020), S263–7210.2967/jnumed.120.25453233293447.

22 On imaging technologies as forms of disciplinary boundary work, see Regula Valérie Burri , ‘Doing Distinctions: Boundary Work and Symbolic Capital in Radiology’, Social Studies of Science, 38, 1 (2008), 35–62 .

23 See, for example, John D. Cockcroft , ‘George de Hevesy. 1885–1966’, Biographical Memoirs of Fellows of the Royal Society, 13 (1967), 125–66; Marshall Brucer , A Chronology of Nuclear Medicine (St. Louis: Heritage Publications, 1990); Sten Carlson , ‘A Glance at the History of Nuclear Medicine’, Acta Oncologica, 34, 8 (1995), 1095–10210.3109/028418695091272368608034; Siegfried Niese , Georg von Hevesy 1885–1966 (Münster: Principal, 2009); and Carolyn J. Anderson , Xiaoxi Ling , David J. Schlyer , and Cathy S. Cutler , ‘A Short History of Nuclear Medicine’, in Jason S. Lewis , Albert D. Windhorst , and Brian M. Zeglis (eds.), Radiopharmaceutical Chemistry (Cham: Springer, 2019), 11–26 .

24

William G. Myers and Henry Wagner , ‘Nuclear Medicine. How It Began’, Hospital Practice, 9, 3 (1974), 103–13; Jerry Obaldo and Barbara Hertz , ‘The Early Years of Nuclear Medicine. A Retelling’, Asia Oceania Journal of Nuclear Medicine and Biology, 9, 2 (2021), 207–1910.22038/aojnmb.2021.55514.1385PMC825551934250151; and Bo-Anders Jönsson , ‘The History of Nuclear Medicine’, in Michael Ljungberg (ed.), Handbook of Nuclear Medicine and Molecular Imaging for Physicists: Instrumentation and Imaging Procedures, vol. 1 (Boca Raton: CRC Press, 2022), 1–14 .

25 See the comprehensive accounts by Michael Feld and Michel De Roo , Geschichte der Nuklearmedizin in Europa (Stuttgart: Schattauer, 2000); Ralph McCready , Gopinath Gnanasegaran , and Jamshed Bomanji (eds), A History of Radionuclide Studies in the UK (Cham: Springer, 2016)29787040; and Paul Richards , Isotopes, Imaging, and Identity. The History of Nuclear Medicine in Australia and New Zealand (Sydney: Australian and New Zealand Society of Nuclear Medicine, 2013). Personal insights are provided, for example, by John Mallard and Nigel Trott , ‘Some Aspects of the History of Nuclear Medicine in the United Kingdom’, Seminars in Nuclear Medicine, 9, 3 (1979), 203–1710.1016/s0001-2998(79)80034-3116364; Rudolf Höfer and Herbert Vetter , ‘Die Nuklearmedizin an der II. Med. Univ.-Klinik’, in Josef Dézy (ed.), Die II. Medizinische Universitätsklinik in Wien (Vienna: Brandstätter, 1994), 48–54 ; Thérèse Planiol , Une femme, un destin (Paris: Éditions Rive Droite, 1996); Henry Wagner , A Personal History of Nuclear Medicine (London: Springer, 2005); and Emilio Bambardii and Savvas Frangos (eds), Nuclear Medicine. Fusing the Ideas of Democritus and Hippocrates (Vienna: European Association of Nuclear Medicine, 2012).

26

Herbert Vetter , ‘Nuclear Medicine – a New Discipline’, IAEA Bulletin, 8, 2 (1965), 7–13 . Foundational textbooks in the field included William Blahd , Franz Bauer , and Benedict Cassen , The Practice of Nuclear Medicine (Springfield: Thomas, 1958), and Norman Veall and Herbert Vetter , Radioisotope Techniques in Clinical Research and Diagnosis (London: Butterworth, 1958).

27 For the early establishment of nuclear medicine institutions in various European countries, see Feld and De Roo, *op. cit.* (note 25), 5.

28 An overview can be found in Montague Cohen , Robert A. Dudley , Godofredo Gómez-Crespo , Joseph D. Pearson , Kia Chi Tsien , and Herbert Vetter , ‘The International Atomic Energy Agency’s Work in the Field of Nuclear Medicine’, Nuklearmedizin, 2, 4 (1962), 435–4713880283.

29 See Gerald Kutcher , Contested Medicine: Cancer Research and the Military (Chicago: University of Chicago Press, 2009); Angela Creager , ‘Nuclear Energy in the Service of Biomedicine: The U.S. Atomic Energy Commission’s Radioisotope Program’, Journal of the History of Biology, 39 (2006), 649–8410.1007/s10739-006-9108-217575955; and Alison Kraft , ‘Manhattan Transfer: Lethal Radiation, Bone Marrow Transplantation, and the Birth of Stem Cell Biology, ca. 1942–1961’, Historical Studies in the Natural Sciences, 39, 2 (2009), 171–218 10.1525/hsns.2009.39.2.17120073126.

30 See, among others, Ronald Doel , ‘Scientists as Policymakers, Advisors, and Intelligence Agents’, in Thomas Söderquist (ed.), Historiography of Contemporary Science and Technolog (London: Routledge, 1997), 215–44; John Krige , American Hegemony and the Postwar Reconstruction of Science in Europe (Cambridge, MA: MIT Press, 2006); Simone Turchetti , Greening the Alliance: The Diplomacy of NATO’s Science and Environmental Initiatives (Chicago: University of Chicago Press, 2018); and Söenke Kunkel (ed.), ‘Science Diplomacy in the Twentieth Century’, Journal of Contemporary History, 56, 3 (2021), 473–562 .

31 See, for example, Gabrielle Hecht , Being Nuclear: Africans and the Global Uranium Trade (Cambridge, MA: MIT Press, 2012); Angela Creager and Maria Rentetzi , ‘Sharing the “Safe” Atom? The IAEA and Nuclear Regulation Through Standardisation’, in Bernadette Bensaude-Vincent , Soraya Boudia , and Kyoko Sato (eds), Living in a Nuclear World (London: Routledge, 2022), 111–31; Maria Rentetzi (ed.), ‘The Politics of Radiation Protection’, NTM Zeitschrift für Geschichte der Wissenschaften, Technik und Medizin, 30, 3 (2022), 125–270 10.1007/s00048-022-00332-zPMC916014735499560; Kenji Ito , ‘The Scientific Object and Material Diplomacy: The Shipment of Radioisotopes From the U.S. to Japan’, Centaurus, 63 (2021), 296–319 .

32 Kraft, *op. cit.* (note 8); María Santesmases , ‘From Prophylaxis to Atomic Cocktail: Circulation of Radioiodine’, Dynamis, 29 (2009), 337–6310.4321/s0211-9536200900010001519852395; Gisela Mateos and Edna Suárez Díaz , ‘Creating the Need in Mexico: The IAEA’s Technical Assistance Programs for Less Developed Countries (1958–68)’, History and Technology, 36, 3–4 (2020), 418–36 ; Leonid Il’in and Aleksandr Samoilov , ‘The Role of Radiobiology and Nuclear Medicine in Protection From the Effects of Ionizing Radiation’, Herald of the Russian Academy of Sciences, 91, 3 (2021), 355–6310.1134/S1019331621030072PMC831970834341648; Ursula Heim , ‘Das medizinische Atom: Radioisotope in internistischen Kliniken der Bundesrepublik Deutschland, 1945 bis 1965’, NTM Zeitschrift für Geschichte der Wissenschaften, Technik und Medizin, 33 (2025), 145–7310.1007/s00048-025-00415-7PMC1221396940358679; and Mattes and Philippe, *op. cit.* (note 14).

33 On medical specialties, see George Weisz , Divide and Conquer: A Comparative History of Medical Specialization (Oxford: Oxford University Press, 2006).

34 On gendered opportunities and exclusions in radioactivity research, medical physics, and nuclear medicine, see Maria Rentetzi , ‘Gender, Politics, and Radioactivity Research in Interwar Vienna: The Case of the Institute for Radium Research’, Isis, 95, 3 (2004), 359–9310.1086/42896015747771; Jill Steuer , ‘Women in Nuclear Medicine’, Journal of Nuclear Medicine, 37, 3 (1996), 15N–19N 8772631; and Laura Evangelista , Agata Pietrzak , Ozgul Ekmekcioglu , Petra Petranovic Ovcaricek , Valentina Ambrosini , Sona Balogova , Lidia Cunha , Amy Eccles , Valentina Garibotto , Valerie Lewington , Ora Israel , and Jolanta Kunikowska , ‘Gender Issues in the Nuclear Medicine Community: Results From a Survey Promoted by the EANM Women Empowerment Task Force’, European Journal of Nuclear Medicine and Molecular Imaging, 49 (2022), 2106–1210.1007/s00259-022-05790-0PMC898380335384461. Women continued to face persistent barriers in many established medical specialities, but the hybrid character of early nuclear medicine – linking technology, laboratory research, and clinical practice – could allow women trained in physics, chemistry, pharmacy, or medicine to move between the natural sciences and clinical settings. Such opportunities were often supported by international training, mentorship, and visible role models such as Irène Joliot-Curie (1897–1956). These openings, however, remained fragile: as nuclear medicine acquired greater institutional recognition, prestige, and funding, women’s participation could decline, reflecting broader patterns of gender exclusion in science and medicine.

35 Néstor Herran and Sebastian Grevsmühl, *The First Nuclear Industry: Radioisotopes, State, and Society* (2017). https://hal.science/hal-01824590/document (accessed on 15 April 2025).

